# The Camel Adaptive Immune Receptors Repertoire as a Singular Example of Structural and Functional Genomics

**DOI:** 10.3389/fgene.2019.00997

**Published:** 2019-10-17

**Authors:** Salvatrice Ciccarese, Pamela A. Burger, Elena Ciani, Vito Castelli, Giovanna Linguiti, Martin Plasil, Serafina Massari, Petr Horin, Rachele Antonacci

**Affiliations:** ^1^Department of Biology, University of Bari “Aldo Moro,” Bari, Italy; ^2^Research Institute of Wildlife Ecology, Vetmeduni Vienna, Vienna, Austria; ^3^Department of Biosciences, Biotechnologies and Biopharmaceutics, University of Bari “Aldo Moro,” Bari, Italy; ^4^Department of Animal Genetics, Faculty of Veterinary Medicine, University of Veterinary and Pharmaceutical Sciences, Brno, Czechia; ^5^CEITEC-VFU, University of Veterinary and Pharmaceutical Sciences, RG Animal Immunogenomics, Brno, Czechia; ^6^Department of Biological and Environmental Science and Technologies, University of Salento, Lecce, Italy

**Keywords:** Immunome, Old World camelids, *Camelus bactrianus*, *Camelus dromedarius*, *Camelus ferus*, Immunoglobulins, T cell receptors, major histocompatibility complex

## Abstract

The adaptive immune receptors repertoire is highly plastic, with its ability to produce antigen-binding molecules and select those with high affinity for their antigen. Species have developed diverse genetic and structural strategies to create their respective repertoires required for their survival in the different environments. Camelids, until now, considered as a case of evolutionary innovation because of their only heavy-chain antibodies, represent a new mammalian model particularly useful for understanding the role of diversity in the immune system function. Here, we review the structural and functional characteristics and the current status of the genomic organization of camel immunoglobulins (IG) or antibodies, α/ß and γ/δ T cell receptors (TR), and major histocompatibility complex (MHC). In camelid humoral response, in addition to the conventional antibodies, there are IG with “only-heavy-chain” (no light chain, and two identical heavy gamma chains lacking CH1 and with a VH domain designated as VHH). The unique features of these VHH offer advantages in biotechnology and for clinical applications. The TRG and TRD rearranged variable domains of *Camelus dromedarius* (Arabian camel) display somatic hypermutation (SHM), increasing the intrinsic structural stability in the γ/δ heterodimer and influencing the affinity maturation to a given antigen similar to immunoglobulin genes. The SHM increases the dromedary γ/δ repertoire diversity. In *Camelus* genus, the general structural organization of the TRB locus is similar to that of the other artiodactyl species, with a pool of *TRBV* genes positioned at the 5’ end of three in tandem D-J-C clusters, followed by a single *TRBV* gene with an inverted transcriptional orientation located at the 3’ end. At the difference of TRG and TRD, the diversity of the TRB variable domains is not shaped by SHM and depends from the classical combinatorial and junctional diversity. The MHC locus is located on chromosome 20 in *Camelus dromedarius*. Cytogenetic and comparative whole genome analyses revealed the order of the three major regions “Centromere-ClassII-ClassIII-ClassI”. Unexpectedly low extent of polymorphisms and haplotypes was observed in all Old World camels despite different geographic origins.

## Introduction

In vertebrates, B and T lymphocytes together with the antigen-presenting cells play central roles in the adaptive immune system. They respond to a large variety of antigens that are specifically recognized through highly specialized proteins: immunoglobulins (IG) or antibodies in B cells, and T cell receptors (TR) in T cells (Lefranc, 2014a). The common shape of IG is the tetrameric structure, two identical dimers each made up of an IG heavy (H) chain, and an IG light (L) chain (either IG light kappa (IGK) or IG light lambda (IGL) chains). The TR is a heterodimeric receptor that may occur in two types: αβ (TR-Alpha_Beta, composed of a T cell receptor alpha (TRA) and a T cell receptor beta (TRB) chain) and γδ (TR-Gamma_Delta, composed of a T cell receptor gamma (TRG) and a T cell receptor delta (TRD) chain).

Each chain contains a variable domain and a constant region (Lefranc, 2014a). The variable domain forms the antigen-binding site and it is generated during B or T lymphocyte development by a sequential gene rearrangement at the DNA level of the *variable (V)* and *joining (J)* genes of the IGK or IGL, TRG, and TRA loci, and *V*, *diversity (D)*, and *J* genes of the IGH, TRB and TRD loci. After transcription, the rearranged V-(D)-J sequence is spliced to the *constant (C)* gene (Lefranc and Lefranc, 2001; Lefranc and Lefranc, 2001b; Jung and Alt, 2004). The resulting IG and TR chains are proteins with a variable (V) domain at the N-terminal end. Each V domain comprises nine beta sheets forming four framework regions or FR, which support three hypervariable loops (complementarity determining regions or CDR) (Lefranc 2014; Lefranc and Lefranc 2019). CDR1 and CDR2 are encoded by the germline V gene; the third, CDR3, results from the V-(D)-J rearrangement. The six CDR loops of the paired V domains of the IG (VH and VL) and those of the TR gamma/delta (V-gamma and V-delta) contribute to the antigen-binding site. In contrast in the TR alpha/beta, only the two CDR3 principally recognize and bind the antigenic peptide bound to major histocompatibility (MH) proteins of class I (MH1) or class II (MH2), whereas the germline-encoded CDR1 and CDR2 loops mainly contact the helices of the MH proteins (Lefranc, 2014a).

For IGH chains, the rearranged variable domain VH will initially be expressed together with IGHM, the most J-proximal *IGHC* gene, leading to the IgM class synthesis. After the encounter with the antigen and B cell activation and with T cell cooperation, a further DNA recombination event, referred to as class switch recombination, can take place in B cells, resulting in replacement of the IGHM by one of the gene of the other IGHC gene subgroups, namely, IGHG, IGHE, or IGHA. This process leads to the expression of a new H chain with different effector functions, thereby shifting the IG from the IgM class to one of the IgG or IgA subclasses or to IgE class (Lefranc and Lefranc, 2001)

The genes encoded for each IG or TR chain are located in different loci. There are three IG loci (IGK, IGL, and IGH) and four TR loci (TRA, TRB, TRG, and TRD) (http://www.imgt.org/IMGTrepertoire/LocusGenes). The TRA and TRD loci occupy the same chromosome location, being the TRD inserted into the TRA locus. The number of the *V*, *D*, and *J* genes within loci as well as their genomic organization can vary significantly among species. This implies that the gene content is an important element in generating the full extent of the IG and TR repertoires, providing the species with the ability to adapt to its own habitat to defend against infections from a large variety of pathogens.

The complex response of camelids to different pathogens has been investigated over nearly three decades. In this focused review, we provide a comprehensive overview based on the search of key publications from the more recent literature on the genomic and functional characteristics of the IG, TR, and MH molecules in camelids.

## The Camel Immunoglobulin: a Dichotomous Adaptive Humoral Immune System

**Figure 1:**
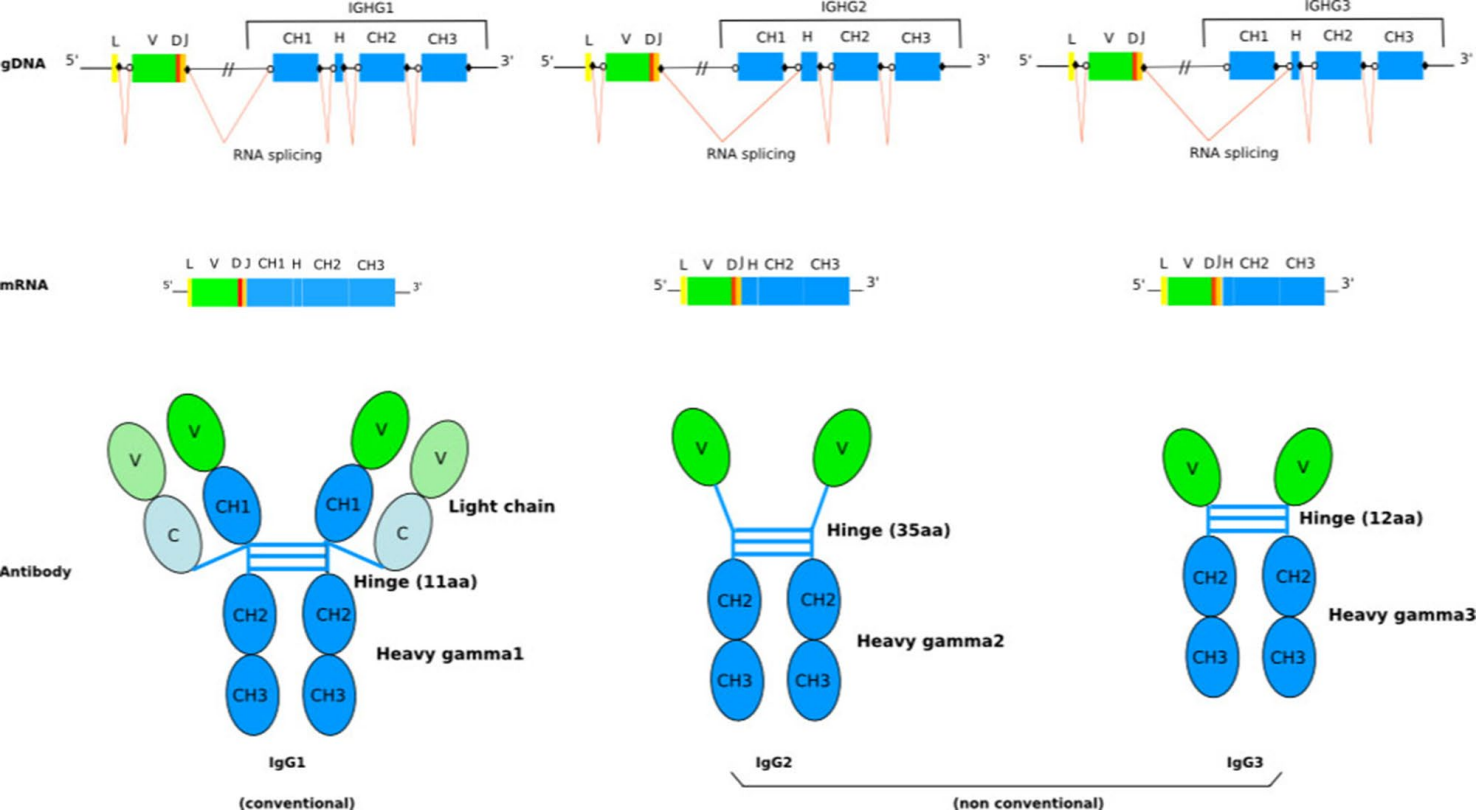
Schematic representation of the general structure of camel conventional IgG1 and the nonconventional IgG2 and IgG3. The lack of light chain is itself a consequence of the absence of the CH1 domain in the gamma 2 and gamma 3 chains, due to a splicing defect of the CH1 exon. The figure is from IMGT: Camelidae IgG antibodies (http://www.imgt.org/IMGTbiotechnology/Camel_IgG.html#characteristics) with permission of IMGT ^®^, the international ImMunoGeneTics information system ^®^ (http://www.imgt.org).

The humoral immune system of camelids (i.e., *Camelus bactrianus* (Bactrian camel), *C. dromedarius* (Arabian camel), *C. ferus* (Wild Bactrian camel), *Lama glama* (llama), *L. guanaco* (guanaco), *Vicugna pacos* (alpaca), and *V. vicugna* (vicugna) have the particularity of including, in addition to the conventional tetrameric IgG (IgG1 subclass) composed of two identical heavy (H) and two identical light (L) chains connected by disulphide bonds, functional homodimeric IgG (IgG2 and IgG3 subclasses) lacking L chains and, therefore, comprising only two identical H chains (only-heavy-chain antibodies hcAb, or hcIG) (**Figure 1**) (Hamers-Casterman et al., 1993). Sequence and structure analysis revealed a number of characteristic features of camelid hcAb (Muyldermans and Lauwereys, 1999) to make the H chain functional in antigen binding in absence of the L chain. Besides their dissimilar IG chain content, tetrameric, and homodimeric IgG display distinct H chains, with the H chain within hcAb composed of three instead of four globular domains. Biochemical and cDNA sequence analyses have shown that the C region (CHH) of the homodimeric IgG lacks the first domain, CH1, which normally binds to the L chain. This region is eliminated by splicing during mRNA processing due to a point mutation on the donor-splicing site present in the first C exon/intron boundary (Nguyen et al., 1999; Woolven et al., 1999). Hence, the variable domain is joined directly to the hinge region in hcAb (Lefranc, 2014b) (**Figure 1**). The hinge region itself can be different from conventional IG heavy chains (Henry et al., 2019)

### Structural Features and Binding Properties of the VHH Domain

Since the hcAb do not contain L chains, the antigen-binding site is reduced to a single domain (referred to as VHH) that resembles the structure of the H chain variable domain (VH) of the tetrameric IgG. However, the VHH domains display remarkable amino acid differences in positions that are conserved in the conventional VH domains. In FR2-IMGT, amino acids highly conserved across species, located at positions 42, 49, 50, and 52, according to the IMGT unique numbering (Lefranc et al., 2003; Lefranc, 2011a; Lefranc, 2011b), which, in conventional VH domains, form the hydrophobic surface associating with VL (Chothia et al., 1985), are changed to more hydrophilic amino acids (Hamers-Casterman et al., 1993; Muyldermans et al., 1994; Vu et al., 1997; Tillib et al., 2014; Li et al., 2016; Brooks et al., 2018). The hydrophobic to hydrophilic amino acid changes at these positions make impossible the association of the VHH with a conventional VL domain and undoubtedly help in the solubility behaviour of the hcAb (Davies and Riechmann, 1994; Muyldermans et al., 1994). Moreover, the amino acid change, in most of the Bactrian and Arabian camel V-REGION, of Leu (L) 12 (IMGT numbering) > Ser (S) in FR1, which is seen as an adaptation to the absence of the CH1 domain, helps the solubility of the hcAb. However, the VHH are notably more conserved in sequence and structure across their FR regions than conventional VH domains (Mitchell and Colwell, 2018b).

CDR1-IMGT and CDR2-IMGT of the IGHV encoding VH or VHH domains are highly similar in term of sequences and lengths (IMGT Repertoire (IG and 1TR) > Protein displays Arabian camel (*Camelus dromedarius*)).^1^

Conversely, the CDR3 loop of the hcAb tends to be longer and more variable than that of the conventional IgG (Muyldermans, 2013; Mitchell and Colwell, 2018a; Mitchell and Colwell, 2018b). This difference is much higher in *Camelus bactrianus* than that reported for llama and dromedary (Henry et al., 2019). A longer CDR3 in the hcAb could potentially greatly increase both the sequence and shape diversity of the paratope. As recently reported (Mitchell and Colwell, 2018a), the CDR3 loop is more frequently in contact with the antigen than CDR1 and CDR2 loops. Therefore, CDR3 plays a more dominant role in determining interaction specificity. Moreover, in Bactrian as well as in dromedary camel, the long rearranged CDR3 of VHH in most cases harbours a cysteine that forms a disulphide bond with another additional cysteine located either in CDR1, or on position 50 (*Camelus dromedarius* (Camdro) IGHV) or 55 (*Lama glama* (Lamgla) IGHV) in FR2-IMGT (IMGT Repertoire> IMGT Protein display)^1^ (Hamers-Casterman et al., 1993; Muyldermans et al., 1994; Vu et al., 1997). This second disulphide bond stabilizes the VHH domain and fixes the long CDR3 loop into an optimal conformation, increasing the affinity for the antigen (Govaert et al., 2012).

The VHH domain of the hcAb is fully capable of antigen binding. It recognizes a broad range of epitopes with high affinity. Moreover, the hcAb repertoire, largely diversified by extensive somatic hypermutation (SHM) involving the variable domains, results in novel and unusual paratopes different from those of conventional IgG (Nguyen et al., 2000; Nguyen et al., 2001). Many VHH domains are competitive enzyme inhibitors since they interact specifically with the active site of enzymes that, in general, is of low antigenicity for the conventional VH-VL domains (Lauwereys et al., 1998).

The unique characteristics of the VHH and their straightforward bacterial expression have made them of particular interest in biotechnological and pharmaceutical applications. In recent years, the industrialization of camel VHH domains (designated as single-domain antibodies or “nanobodies” for their format) has produced a great expansion of their use (Muyldermans et al., 2009; Hassanzadeh-Ghassabeh et al., 2013; Helma et al., 2015; Fernandes, 2018), and in 2018, a diabody of two VHH humanized from *Lama glama*, caplaximab, an anti-von Willebrand factor (VWF) A1 domain, has been approved by the European Medicine Agency (EMA) in Europe and by the Food and Drug Administration (FDA) in 2019 for treatment of acquired thrombotic thrombocytopenia purpura (TTP) [IMGT/mAb-DB, (Lefranc et al., 2015)]. Beyond their application as therapeutics to treat human diseases (Muyldermans, 2013; Rissiek et al., 2014; Steeland et al., 2016), nanobodies have become a valuable research tool. For example, they are used as affinity reagents to assist the crystallization process, to detect antigen trafficking inside living cells, to interfere with protein–protein interactions, and to direct proteins to degradation (Loris et al., 2003; Hassanzadeh-Ghassabeh et al., 2013; Helma et al., 2015; Beghein and Gettemans, 2017; Baudisch et al., 2018; Schumacher et al., 2018).

### The Camel IGH Locus

To understand the molecular mechanisms governing the formation of tetrameric and homodimeric IgG in camelids, the characterization of the organization of the genes that encode them is an essential step. Although hcAb have been extensively investigated, to date, there has not been a comprehensive analysis of the repertoire based on high-throughput sequencing (Jirimutu et al., 2012; Li et al., 2016; Ali et al., 2019; Henry et al., 2019), but most efforts have been based upon low throughput sequence analysis, and the reports trying to analyse and describe the complete immune repertoire of camel hcAb are limited. Although the annotated data in public databases are limited, the available sequences show that the camelid *IGHV* genes, which encode VH and VHH, belong to the IGHV3 subgroup (IMGT Repertoire (IG and TR) 2. Proteins and alleles > Protein displays Arabian camel (*Camelus dromedarius*); ibid:alpaca (*Vicugna pacos*) IGHV; and ibid:llama (*Lama glama*) IGHV)^1^. The high percentage of identity between IGHV encoding VH or VHH classifies them in the same IGHV3 subgroup, the differences between them being the characteristic amino acid changes at the four IMGT positions 42, 49, 50, and 52. It is, therefore, the IGHV, which is involved in the rearrangement which determines if the expressed domain is VH or VHH. The constant region of the *Camelus dromedarius* H-gamma1 chains is encoded by the *IGHG1* gene, whereas the constant region of the H-gamma2 and H-gamma3 chains are encoded by *IGHG2* and *IGHG3* genes, which both have a splicing defect of the CH1 DONOR-SPLICE leading to the absence of the CH1 in the transcript, although this CH1 sequence is present in the genomic DNA (**Figure 1**) (Lefranc, 2014b) (IMGT Biotechnology > Antibody camelization > Characteristics of the camelidae (camel, llama) antibody synthesis)^1^. The presence of a point mutation (G to A) in the putative donor splicing site flanking the first C exon and the specific hinge region makes it possible to distinguish the *IGHG* genes encoding the constant region of hcAb chain and the *IGHG* genes encoding the constant region of conventional antibody heavy chain (Vu et al., 1997; Nguyen et al., 1999; Woolven et al., 1999). In Arabian camel, three *IGHG* genes have been identified (IMGT Repertoire (IG and TR) > Gene table > Gene table: Arabian camel (*Camelus dromedarius*) IGHC)^1^. Four *IGHG* genes (*IGHG1A*, *IGHG1B*, *IGHG2B,* and *IGHG2C*, these last two genes without CH1 in the alpaca genome) are present in the alpaca and llama genomes (Achour et al., 2008; Henry et al., 2019) (IMGT Repertoire (IG and TR) > Gene table > Gene table: Alpaca (Vicugna pacos) IGHC)^1^, even if other studies have also identified *IGHG2A* and *IGHG3* genes in the expressed IgG repertoire of llama (De Genst et al., 2006; Saccodossi et al., 2012).

*IGHV* genes encoding VH and VHH domains as well as the *IGHG* genes encoding the C-REGION of conventional IG (IGHG1) and only-heavy-chain IG (IGHG2 and IGHG3) are in an intermixed conformation, in V and C clusters, respectively, in a single IGH locus (**Figure 1**). *IGHV* genes encoding VH and VHH domains recombine with the same *IGHD* and *IGHJ* genes. The identification of identical *IGHD* and *IGHJ* genes in VH and VHH cDNAs suggests the common use of the *IGHD* and *IGHJ* genes (Harmsen et al., 2000; Nguyen et al., 2000; Achour et al., 2008). The *IGHV* genes are designed in three or four subgroups based on their degree of homology with human IGHV subgroups. There are IGHV subgroups that contain exclusively classical *IGHV* genes, whereas one subgroup, IGHV3, contains both classical *IGHV* genes and *IGHV* genes with the FR2-IMGT camelid hydrophilic amino acid in 50 (together with 42, 49, and/or 52) (Lefranc and Lefranc, 2019), which are rearranged and expressed in VH and VHH domains, respectively (Harmsen et al., 2000; Achour et al., 2008; Deschacht et al., 2010; Griffin et al., 2014). The transcription of a VHH domain (V-D-J-REGION) with the IGHG2 or IGHG3 constant region leads to the synthesis of a heavy chain without CH1, which is the characteristic feature of the hcAb repertoire (Hamers-Casterman et al., 1993) (**Figure 1**). However, it has been reported that also classical *IGHV* genes can contribute to the hcAb pool. In this case, the V domain has a classical short junction without the additional cysteine (compared to the VHH) and the conserved anchor Trp 118 is substituted mostly by an arginine codon (Deschacht et al., 2010). It was shown that also the IGHV4 subgroup contributes to produce both classical IgG and hcAb. Interestingly, a same IGHV4-IGHD-IGHJ rearrangement has been shown to be shared between a classical tetrameric IgG and a dimeric hcAb (heavy chain with no CH1, and no light chain).

From a limited number of germline *VHH* genes, camelids can generate a large and diversified repertoire by extensive SHM (Nguyen et al., 2000). The pattern of variability particularly within CDR loops but also in framework regions is larger in VHH than in VH cDNAs. As for classical VH, crystallographic studies of the VHH-antigen complexes demonstrated that amino acids located in the CDR1 and CDR2 loops and, particularly, the long CDR3 interact with the antigen (Desmyter et al., 1996; Decanniere et al., 1999). Contact analysis between the VHH and the ligand is provided in IMGT/3Dstructure-DB, in 38 entries of *Camelus dromedarius* and 58 for *Lama glama* (August 2019). Thus, the introductions of mutations together with a long CDR3 increase the VHH potential repertoire for antigen binding.

## Dromedary *TRG* and *TRD* Genes

### The Genomic Organization of the Dromedary TRG Locus

γδ T cells have unique features when compared with the more abundant αβ T cells, e.g., a preferential distribution in both epithelial and mucosal sites, and an immunoglobulin like antigen recognition mechanism in addition to the MH-restricted one. In the immune response during inflammatory processes, γδ T cells release cytokines and kill infected macrophages; they combine the characteristics of an innate-like immune response with those of an adaptive response to inflammation (Allison et al., 2001; Allison and Garboczi, 2002; Adams et al., 2005). Their percentage in peripheral blood cells, depending on age and species, differs strikingly from that of αβ T cells (Carding and Egan, 2002). Artiodactyls (sheep, cows and pigs) are referred to as “γδ-high species” since they exhibit a higher frequency and a wider physiological distribution of γδ T cells with respect to other mammalian species, including humans and mice, which are referred to as “γδ-low species” (Hein and Dudler, 1993; Ciccarese et al., 1997).

Recent studies have shown the presence of SHM in the γ chain of gamma/delta in shark T cells and in both chains γ and δ of the dromedary camel (Chen et al., 2009; Antonacci et al., 2011; Chen et al., 2012; Vaccarelli et al., 2012). In each work, it was shown that SHM followed the characteristics of the mutational profiles detected in the B cells that undergo affinity maturation. γδ T cells, unlike αß T cells, interact with nonclassical major histocompatibility complex (MHC) and have a small number of genes, so they have a limited diversity (Allison et al., 2001; Adams et al., 2005). The SHM seems to be used as a mechanism for further diversification of the γδ receptor and for an optimal recognition of the ligand. Consequently, this would allow the evolutionary changes in the loci, which, in turn, allow the receptor to evolve more rapidly in mutant environments (Adams et al., 2005; Kazen and Adams, 2011).

In “γ/δ-high” species, the TRG and TRD expressed repertoire is mainly affected by a large number of genes distributed in reiterated duplications of functional TRG cassettes (Vaccarelli et al., 2005; Conrad et al., 2007; Vaccarelli et al., 2008) and by a marked expansion and preferential usage of the TRDV1 multigene subgroup (Ishiguro et al., 1993; Yang et al., 1995; Hein and Dudler, 1997; Massari et al., 2000; Antonacci et al., 2005). Usually, in mammals, less than a few exceptions such as in human (Lefranc and Rabbitts, 1989) and in dolphin (Linguiti et al., 2016), TRG loci are quite complicated, containing numerous *V*, *J*, and *C* genes, sometimes located in different chromosomal bands (Massari et al., 1998; Miccoli et al., 2003), or spanning hundreds of kb (Massari et al., 2009).

In *Camelus dromedarius*, the TRG locus spans approximately 45 kb and it maps in a homology region established between bovids chromosome 4, human chromosome 7, and pig chromosome 9, where orthologous TRG loci have been mapped (Vaccarelli et al., 2012).

The dromedary locus consists of two *TRGV* genes (*TRGV1* and *TRGV2*), four *TRGJ* genes, and two *TRGC* genes (*TRGC1* and *TRGC2*), all in the same transcriptional orientation, organized in two functional cassettes (5’-TRGV1-TRGJ1-TRGJ1-2-TRGC1 and TRGV2-TRGJ2-1-TRGJ2-2-TRGC2 -3’). Considering the exon organization of the ovine and human C regions, we inferred that both the dromedary C regions keep a connecting region encoded by three different exons, as is observed in the sheep *TRGC2*, *TRGC4*, and *TRGC6* genes (Miccoli et al., 2001) and in the polymorphic human *TRGC2* gene (Lefranc and Lefranc, 2001b). The dromedary locus organization in two (V-J-J-C) cassettes potentially limits the combinatorial usage of its genes. However, cDNA sequencing clearly revealed that, besides the combinatorial diversity and the introduction of N region diversity typical of all known *IG* and *TR* genes (Lefranc and Lefranc, 2001a; Lefranc and Lefranc, 2001b), the SHM mechanism enhances the TRG and TRD repertoire diversity in *Camelus dromedarius* (Ciccarese et al., 2014).

### SHM in TRG and TRD V Domains and Nature of AA Changes

Among mammals, SHM occurs primarily in germinal center B cells, it introduces point mutations into the variable domains of IG, and it is the driving force for antibody affinity maturation (Li et al., 2004). During SHM, the dgyw/wrch motif (where d = a or g or t, y = c or t, w = t or a, r = a or g, and h = c or t or a) has been found to be the principal hotspot for activation cytidine deaminase (AID) inducing g:u lesions in rearranged *IG* genes (Rogozin and Diaz, 2004; Liu and Schatz, 2009). These changes are dominated by point mutations and biased toward transitions (G:A and C:T). Moreover, the principal site for a/t mutations has been identified in the dinucleotide target wa/tw. This secondary mutator has allowed to define the roles of the error-prone polymerases in mismatch repair (Pavlov et al., 2002; Zhao et al., 2013).

The features of mutations were evaluated comparing the genomic sequence of the single *TRDV4* gene with the TRDV4 cDNA clones derived from spleen and blood, excluding CDR3 and including the TRDJ4 genomic sequence. Four related sets of TRDV4 clones with common CDR3 sequences and unrelated sequences derived from independent rearrangements in blood and spleen were deduced (Antonacci et al., 2011). The analysis of mutations identified a clonal genealogy among related sequences, showing that tandem mutations resulted from sequential point mutations and, given the TRDV4 gene uniqueness, the nucleotide substitutions are not the result of gene conversion events (Antonacci et al., 2011). AID-dependent deamination of cytidine to uracil produces mutations at c/g nucleotides activates the repair proteins MSH2-MSH6 to bind u:g mismatches and recruits the lowfidelity DNA polimerase η (Pol η) (Wilson et al., 2005; Schanz et al., 2009; Zhao et al., 2013). The analysis of substitutions in the TRDV1 and TRDV4 mutated sequences show a bias for transition changes in blood and spleen. Therefore, the nature of V domain resulting from dromedary *TRG* and *TRD* rearranged genes is the result of a combined action of AID, uracil-DNA glycosylase (UNG), and mismatch (MSH) repair pathways (**Supplementary Figure 1**) (Vaccarelli et al.2012; Ciccarese et al., 2014).

In mutated TRGV1 and TRGV2 clones, replacement mutations occur preferentially in bases inside the [dgyw] and [wrch] AID motifs, whereas neutral mutations are favoured outside the motifs. Chi-square analysis used to compare observed and expected numbers of replacement mutations between FR and CDR regions highlighted no significant difference in TRGV1 and in TRDV4 clones. On the contrary, in TRGV2 and TRDV1 clones, the difference in the R/S ratio between CDR and FRs was significant. This feature is consistent with a selection pressure acting on dromedary γδ T cells and with the affinity maturation during clonal expansion.

**Figure 2:**
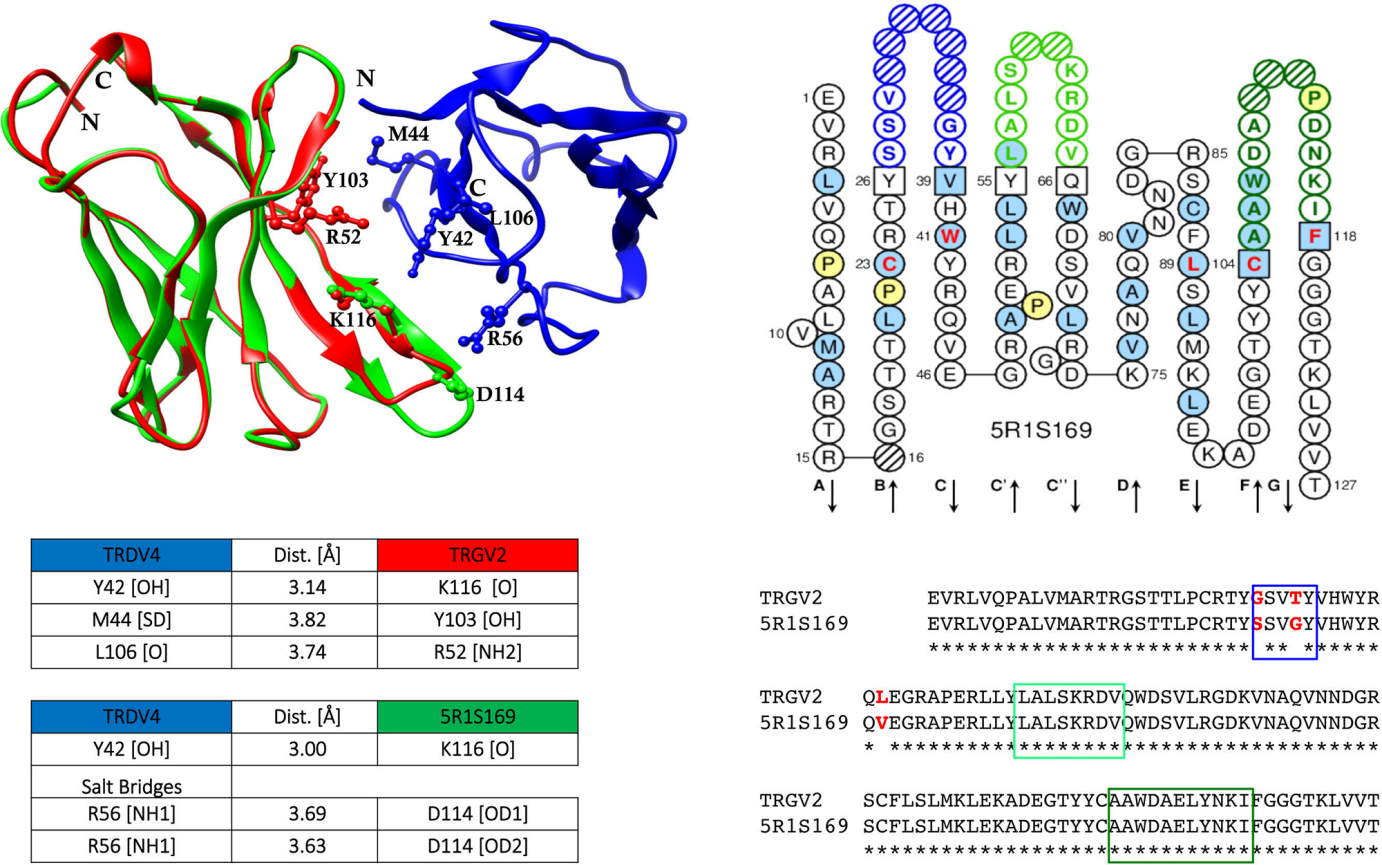
Sequence comparison of dromedary TRGV2/5R1S169, and the computed interaction between the genomic TRGV2, its mutated counterpart 5R1S169, and the genomic TRDV4 (modified from Ciccarese et al., 2014). Structural overlapping of TRGV2 (depicted in red), and 5R1S169 clone (depicted in green) with TRDV4 (depicted in blue), is shown. CDR-IMGT regions are framed with colored rectangles according to IMGT standardized colors. IMGT Collier de Perles of 5R1S169 clone is shown (Lefranc et al., 2003; Lefranc, 2011a; Lefranc, 2011b). The protein complex interface was computed by the online tool PDBePISA at the EBI server. (http://www.ebi.ac.uk/msd-srv/prot_int/) and visualized by UCSF Chimera tool (http://www.cgl.ucsf.edu/chimera/).

When shared mutations were used as position set-points to construct putative lineages from TRG and TRD cDNA clone sets, each of them sharing an identical CDR3, the inferred lineages harbouring multiple radiations highlighted that the substitutions that arose first (progenitor mutations) were replacements changes. These progenitor mutations occurred starting from the CDR3/FR4 region and proceeding toward the leader region along the variable domain with additional mutations in FR2 and FR3. Moreover, it was found that progenitor mutations are selected and transmitted during clonal expansion and they are nonconservative of the amino acid physicochemical properties, i.e., change nonhydrophobic amino acid residues to hydrophobic ones. A computed model was constructed with the TRGV2 translated sequences of the corresponding mutated cDNAs; these sequences were visualized in their two-dimensional structure with the IMGT tool Collier de Perles (Ehrenmann et al., 2011; Lefranc, 2011a; Lefranc, 2011b). The comparative modelling procedure was applied using the counterpart γδ human T cell receptor subunits (Ehrenmann et al., 2010; Xu et al, 2011) and a notable difference between the genomic paired TRGV2/TRDV4 and the mutated cDNA TRGV2 paired to genomic TRDV4 was observed (**Figure 2**). Only for the last paired V domains, the occurrence of putative hydrogen bonds and salt bridges confirmed that the changes alter the conformation of the variable domains of the γδ receptor with consequent effects on its stability.

If the replacements of hydrophilic amino acid residues with hydrophobic ones are maintained and positively selected during the proliferation of T cells, it follows that they stabilize the structure of the receptor whether they fall into the CDR or into the FR. Therefore, conclusions are consistent both with the acquisition of new antigenic specificities and repertoire diversification and simultaneously with selection for changes in paratopes in the manner similar to that of immunoglobulin gene during the B cells affinity maturation to a given antigen. The same conclusions were recently reached by Ott et al., in a paper where the authors propose that the SHM in TRA chain contributes to selection of αß T cells in nurse “couch potato” shark thymus (Ott et al., 2018). The absence of SHM for the TRB chain reported recently in expression assays of spleen in dromedary (Antonacci et al., 2017a) could constitute the watershed between the cartilaginous fish that are the most divergent jawed vertebrate group relative to mammals, and mammals in the scenario of the thymic selection of αß and γδ T cells.

In the dromedary TRG and TRD loci, evolution allowed the SHM to increase the receptor repertoire of cell-mediated immunity. Previously, we have proposed that requirements related to immunoprotective functions, including the first defensive barrier in the epithelia of the digestive tract, are likely to have induced in TRG and TRD loci of ruminants a sort of genome functional fluidity resulting in duplications of *TRG* gene cassettes and in a marked expansion of the *TRDV1* multigene subgroup (Vaccarelli et al., 2012).

In this review, we point out that, in dromedary, TRG and TRD evolution was favoured by mutation in the productively rearranged *TRG V-J* and *TRD V-D-J* genes, so that a large and diversified TRG and TRD repertoire could be generated even in absence of functional reiterated gene duplications. Because SHM has not been shown to occur in any mammalian organism, we can hypothesize that Camelidae by themselves might occupy a peculiar immunological niche, proposing the camel lineage as a fascinating model in the evolution of immune systems.

## The Organization and Evolution of *Camelus* TRB Locus Is Shared in Tylopoda, Suina, and Ruminantia

**Figure 3:**
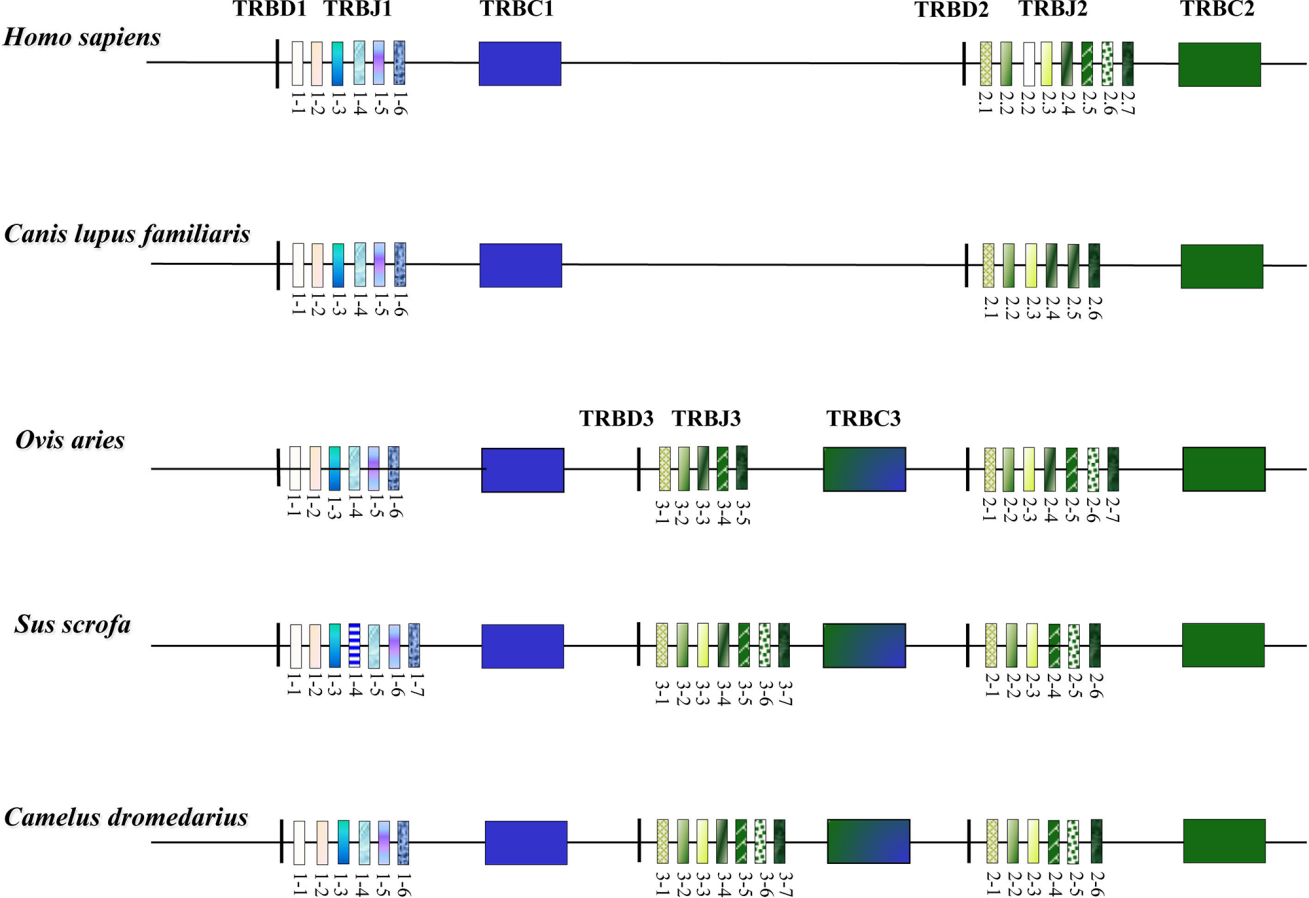
Schematic representation of the D-J-C clusters within the TRB locus of the different mammalian species modified from Antonacci et al., 2017a. The orthologous and paralogous *TRBJ* genes are depicted with the same colour as deduced by the phylogenetic analysis. The double colour *TRBC3* gene indicates the homologies with the *TRBC1* and *TRBC2* genes as result of the unequal crossover that generated the new TRB D-J-C cluster in the artiodactyl species (Antonacci et al., 2008; Eguchi-Ogawa et al., 2009).

The organization of the TRB locus has been extensively investigated in different mammalian species and it consists of a general structure with a group of *TRBV* genes located at the 5’ end of the locus followed by in tandem TRB D-J-C clusters. A *TRBV* gene, with an inverted transcriptional orientation, lies at the 3’ end of the region. A common aspect to most species, such as humans, rabbits, and dogs (Mineccia et al., 2012; Antonacci et al., 2014; Lefranc et al., 2015), is the presence of two TRB D-J-C clusters, each composed of one *TRBD*, several *TRBJ*, and one *TRBC* genes. Instead, three TRB D-J-C clusters composed the TRB locus in artiodactyl species, i.e., sheep, cattle, and pig (Antonacci et al., 2008; Connelley et al., 2009; Eguchi-Ogawa et al., 2009; Massari et al., 2018). The additional TRB D-J-C cluster 3 is located between the conserved TRB D-J-C cluster 1 and 2 (**Figure 3**). The sequence analysis revealed that the new TRB D-J cluster is correlated to the last one, whereas the *TRBC3* gene is more similar to the *TRBC2* gene in the first part and to the *TRBC1* gene sequence in the last part.

The structure of the TRB locus has been recently investigated in *Camelus dromedarius* (Antonacci et al., 2017a; Antonacci et al., 2017b) and in its wild and domestic Bactrian camel congeners, *Camelus ferus* and *Camelus bactrianus* (Antonacci et al., 2019). The analysis showed that the camel TRB organization is similar to that of the other artiodactyl species, with the presence of three TRB D-J-C clusters (**Figure 3**). This outcome suggests that the TRB genomic organization with three TRB D-J-C clusters was established, prior the Tylopoda/Ruminantia/Suina divergence, through a duplication event due to an unequal crossing-over between the ancestral *TRBC1* and *TRBC2* genes. Following duplication, subsequent species-specific diversifications were made that led to the current genomic organization of the 3’ end of the TRB locus in the different artiodactyl species.

As in all mammalian species, *MOXD2* and *EPHB6* genes border the camel locus at the 5’ and 3’ end, respectively, whereas *TRY* genes are interspersed among *TRBV* genes and arranged in two distinct genomic positions (Antonacci et al., 2017a; Antonacci et al., 2017b; Antonacci et al., 2019).

An expression assay conducted on dromedary spleen T cells (Antonacci et al., 2017a) has demonstrated that all the three TRBD-J-C clusters are used to generate a functional TRβ chain increasing the combinational and junctional diversity of the CDR3 domain. Moreover, the analysis of the cDNA collection shows a preferential usage of the *TRBD1* gene followed by the TRBD3 and TRBD2. This may result in a greater efficiency of the PDß1 promoter with respect to the PDß3 and PDß2, whereas the activity of two similar PDß3 and PDß2 could be correlated with their position from 5’ to 3’ within the locus. Furthermore, a prominent utilization of the *TRBJ3* gene set with respect to the TRBJ2 and TRBJ1 clusters was also observed, probably depending on the number of genes that lie in the genomic region. Probably, multiple 12 bp spacer-recombination signal sequence (12-RS) located in a restricted region may increase the local concentration of the RAG protein that mediates the recombinant process (Di Tommaso et al., 2010). Beside the number of *TRBD* and *TRBJ* genes, other mechanisms seem to increase the dromedary TRB chain functional repertoire, including the incorporation of two *TRBD* genes in the rearrangement process, the intercluster recombination, and the trans-rearrangement (Antonacci et al., 2017a).

While the structure of the TRB D-J-C clusters is similar to the other artiodactyl species, the 5’ end of the camel TRB locus appears to be different with a contraction of the total number of the TRBV genes link to a reduction of duplicated events within the TRBV cluster (Antonacci et al., 2017a; Antonacci et al., 2017b; Antonacci et al., 2019). 30 genes in *Camelus ferus* and 33 in *Camelus dromedarius* as in *Camelus bactrianus*, in all cases assigned to 26 different subgroups, are a low number when compared to the 134 *TRBV* genes for bovine, 67 for human and 74 for rabbit, but only slightly lower than that of pig (38 *TRBV* genes) and dog (37 *TRBV* genes) (Connelley et al., 2009; Mineccia et al., 2012; Antonacci et al., 2014; Lefranc et al., 2015; Massari et al., 2018).

**Figure 4:**
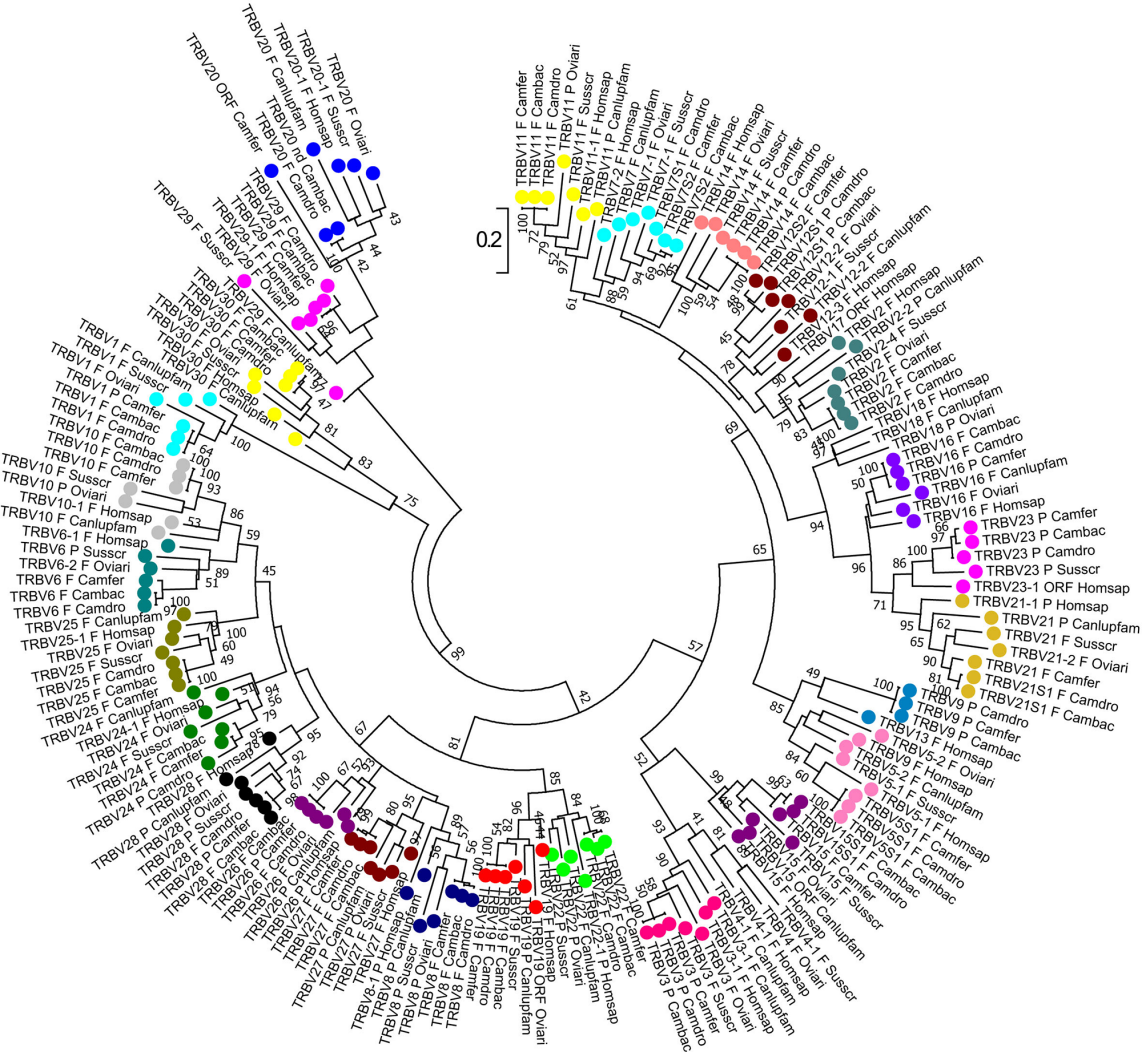
Phylogenetic tree of the dromedary, wild, and domestic Bactrian camel, human, dog, sheep, and pig *TRBV* gene sequences. The sequences of the human, dog, sheep, and pig *TRBV* genes were retrieved from the IMGT database (http://www.imgt.org/), whereas the camel *TRBV* gene sequences derived from Antonacci et al., 2019. The nucleotide sequences of the V-REGION were combined by adopting two selection criteria: (1) only potential functional and in-frame pseudogenes (excepted for human TRBV1) were included; and (2) only one gene for each of the subgroups was selected for each species. The evolutionary analysis was conducted in MEGA7 (Kumar et al., 2016). The evolutionary history was inferred by using the Maximum Likelihood method based on the Tamura-Nei model (Tamura and Nei, 1993). The tree is drawn to scale, with branch lengths measured in the number of substitutions per site. The analysis involved 181 nucleotide sequences. Codon positions included were 1st+2nd+3rd+Noncoding. All positions containing gaps and missing data were eliminated. The different colours highlight the distribution of the phylogenetic groups corresponding to the 26 camel TRBV subgroups. The gene functionality according to IMGT rules (F, functional; ORF, open reading frame; P, pseudogene) is indicated; nd indicates that the nucleotide sequence of the gene is incomplete and its functionality cannot be defined. The IMGT 6-letter for species (Homsap, Susscr, Oviari, Camdro, Camfer, and Cambac) and 9-letter for subspecies (Canlupfam) standardized abbreviation for taxon is used.

The phylogenetic analysis of the *TRBV* genes (**Figure 4**) shows that each of *Camelus ferus*, *Camelus bactrianus*, and *Camelus dromedarius* subgroups come together and form a monophyletic group with a corresponding *TRBV* gene in human and, if present, in dog, sheep, and pig. This is consistent with the occurrence of distinct subgroups prior to the divergence of the different mammalian species. Three human TRBV subgroups (TRBV4, TRBV17, and TRBV18) are lacking in all three camel species, indicating that these subgroups have been lost in these species (i.e., TRBV4) or alternatively they might have originated after the separation of Camelidae (i.e., TRBV18) or Artiodactyla (i.e., TRBV17) from the other mammalian species.

## The MHC in Camels

### The Overall Organization and Genetic Diversity

The MHC has evolved as part of adaptive immunity in vertebrates. In mammals, it spans approximately 4 Mbp and harbours genes, encoding hundreds of proteins with different immune as well as nonimmune functions. The mammalian MHC locus is a complex genomic region that evolved from an ancestral MHC locus, encoding primarily antigen presenting molecules, which are expressed on the surface of the cell and are involved in the immune system’s defence to recognize foreign (“nonself”) substances.

Two classes of antigen-presenting molecules and their genes can be distinguished. MH class I molecules present antigenic peptides originating from self as well as nonself (e.g., virus-encoded) intracellular proteins. MH class II proteins present peptides derived from extracellular proteins (e.g., bacterial products), which were internalized by specialized cells of the immune system. While molecules encoded by MH class I and II genes are mainly responsible for antigen presentation to T lymphocytes (adaptive immunity), the MH class III region includes multiple genes involved among others in the innate immune system, such as tumour necrosis factor alpha (*TNFA*) and members of the complement cascade, which help to eliminate invading pathogens (Trowsdale and Knight, 2013).

**Figure 5:**
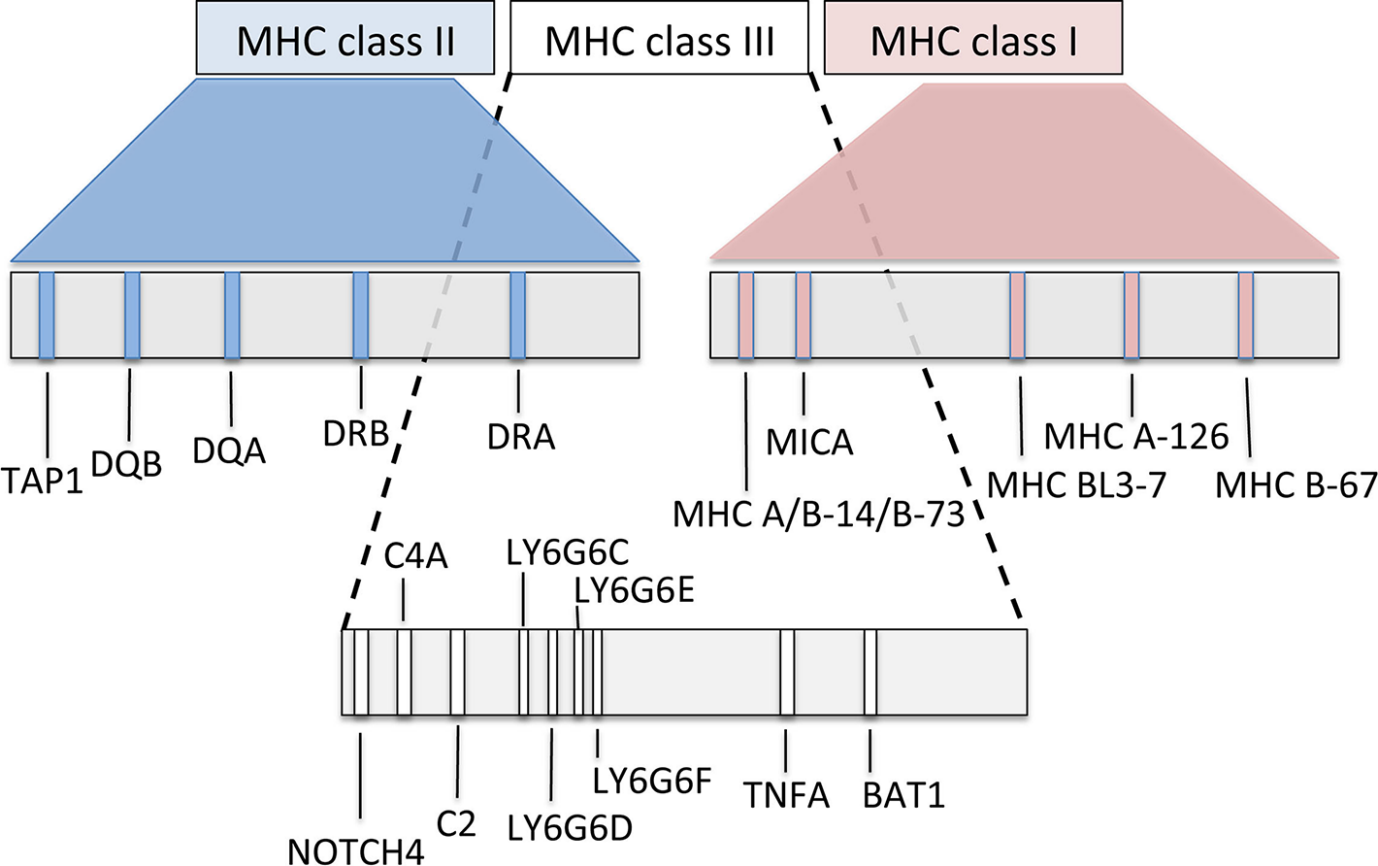
Schematic map of the overall organization of the major histocompatibility complex in Old World camelids, modified from Plasil et al. (2016, 2019).

In Old and New World camelids, the MHC is positioned on the long arm of chromosome 20 (Avila et al., 2014; Plasil et al., 2016). In Old World camels, available genome sequence data (Wu et al., 2014; Fitak et al., 2016a; Fitak et al., 2016b) were combined with initial next generation sequencing to characterize the overall MHC organization (**Figure 5**). The most common general structure of the mammalian MHC region, represented by the order of genes MH class II – MH class III – MH class I, was also confirmed in camels (Plasil et al., 2016; Plasil et al., 2019). Based on the nucleotide sequences retrieved, the MHC of camels seems to be, at least in some of its subregions, more similar to the human and pig MHC rather than to cattle, a phylogenetically closely related species. Besides differences in the location of the *TAP1* gene, phylogenetic analyses of individual MHC genes showed that certain MHC subregions of camels are also closer to pigs than to cattle. Detailed phylogenetic analyses can be found in Plasil et al., 2016 for MH class II and in Plasil et al., 2019 for MH class I.

MH class I and II molecules are heterodimers consisting of two polypeptidic chains (α and ß). While both α and ß polypeptides of the MH class II heterodimers are encoded within the MHC, the MH class I β-2-microglobulin is encoded by a gene located on a different chromosome. In camels, it is on chromosome 6. MH class I loci can be distinguished into “classical” and “nonclassical” MH class I genes, the former coding for antigen-presenting molecules, thus being highly polymorphic, whereas the latter less polymorphic genes code for a group of structurally related proteins with a variety of immune functions. Some of them may even be considered as part of the innate immune system (Allen and Hogan, 2013). MH class II genes are represented by groups of often duplicated loci (e.g., DR, DB, DM, DO, DP, and DQ). Within such a group, *A* genes (e.g., *DRA*) encode the alpha chain and *B* genes (e.g., *DRB*) the beta chain of the class II heterodimer. Most of functionally relevant polymorphisms are concentrated in the antigen binding sites (ABS) of the molecule, encoded by MH class I exons 2 and 3 and by exon 2 of class II genes.

### Overview of the *MHC* Genes Polymorphism

The most characteristic feature of the MHC genes is their high polymorphism, i.e., high numbers (up to hundreds) of allelic variants, especially for genes encoding antigen-presenting molecules. In fact, the MHC is one of the most polymorphic regions in the genome (Janeway et al., 2001). This is important for the immune system to respond fast to rapidly evolving pathogens, a mechanism also described as an evolutionary “arms race” (Sironi et al., 2015).

The genetic diversity of the MHC of camels was first studied in exon 2 sequences of selected class II genes. A surprisingly low level of polymorphism of the *DRA*, *DRB*, and *DQB* genes was observed in all three Old World camel species (Plasil et al., 2016). For the *DRA* locus, *DRA exon 2* spanning 246 bp contains one synonymous and one nonsynonymous single nucleotide polymorphism (SNP) combined in three different alleles shared between dromedaries, domestic and wild Bactrian camels. Successful amplification of this gene in ancient (13^th^ - 16^th^ century, common era) dromedary specimens resulted in three additional substitutions when compared to the reference sequence (Plasil et al., 2016). The *DRB exon 2* (270 bp) contains five polymorphisms shared between the three Old World camel species. In *DQA exon 2,* 11 SNPs, 4 of them synonymous, were identified in *Camelus bactrianus*, of which nine were shared with *Camelus ferus*. In total, three haplotypes were detected, one of them common to all three species and another one shared only between domestic and wild Bactrian camels. The remaining allele was found only in domestic Bactrian camels. The *DQB exon 2* locus was the most polymorphic with 21 polymorphic sites identified across the Old World camels (Plasil et al., 2016). The *DQB* exon 2 harbours a 12-bp long potentially functional insertion not observed in other mammalian species. Since a complete *DQB* exon 2 sequence was not retrieved, the overall extent of polymorphism in this locus remains undefined. However, data available so far suggest that only limited numbers of haplotypes may exist, similarly to the *DQA* locus containing comparable numbers of SNPs. The *DQB* locus is therefore still under investigation.

Similar observations of low diversity were made for MH class I and related loci. In the *classical locus B-67*, only one synonymous polymorphism was found in the entire exon 2 - 3 region. This SNP is shared between dromedaries and Bactrian camels. The *BL3-*7 gene is a locus of unclear status, highly similar to the annotated sequence of the *BL3-6* in alpacas (Avila et al., 2014). Interestingly, it is also closely related to the locus *SLA-11* in pig, one of MHC loci with unknown function and unusual structure. In the Old World camels, this gene contains four SNPs (Plasil et al., 2019). The *MH class I related locus MR1* is an antigen-presenting molecule contributing to the regulation of the microbiome in the intestinal tract (McWilliam et al., 2016). Over the total 22 kbp long *MR1* sequence (located on chromosome 21), 170 polymorphic sites were identified, 5 of them located in the coding sequence and partially shared between dromedaries and Bactrian camels (Plasil et al., 2019). The *MH class I related locus MICA* functions as a stress signalling molecule recognized by the NKG2-D type II receptor on natural killer (NK) cells, αβ T-cells, and γδ T-cells (Shafi et al., 2011; Xiao et al., 2015). A total of 40 SNPs were observed in this sequence, of which eight were found in the coding region (Plasil et al., 2019). It is rather unusual that these MH class I related loci are more polymorphic than a classical MH class I locus, *B-67*. However, knowledge on MH class I classical genes is rather limited and a better-supported conclusion on their diversity can be done only after an extensive analysis of the entire subregion. The first step toward such an analysis and toward an analysis of the MH class III subregion will be made by annotating further genes in a new genome assembly of the dromedary.

## Conclusion

The Camelidae species occupy an important immunological niche within the humoral as well as cell mediated immune response. In additional to the conventional IG, the serum contains a significant amount of IgG composed solely of paired H chains, which are largely diversified by extensive SHM, resulting in novel paratopes different from those of conventional IgG. The antigen binding fragment of these unique hcAbs comprises only one single domain. When produced by microbial expression system, these recombinant miniature antigen binding fragments possess beneficial biophysical properties useful as research tools and for *in vivo* pharmacological applications as candidate drugs for the treatment of human diseases (Muyldermans, 2013; Rissiek et al., 2014; Steeland et al., 2016).

Moreover, an SHM mechanism in productively rearranged *TRD* and *TRG* genes never identified in mammalian species so far, increases the repertoire diversity of the dromedary γδ T cells that recognize the antigen in a manner antibody like. In this contest, the structural changes within the γδ heterodimer, which is stabilized by mutations both in FR and in CDR in genealogical related clones, could enable the acquisition of new antigenic specificity and, at the same time, could influence the affinity maturation to a given antigen in a manner similar to that of *IG* genes (Ciccarese et al., 2014).

As single-chain antibodies and SHM in *TR* genes were described also in cartilaginous fish (Chen et al., 2009; Flajnik et al., 2011; Chen et al., 2012; Ott et al., 2018). It can be argued that a molecular convergence of the adaptive immune response between these species does exist.

Conversely, in αß T cells, the limited germline TRBV repertoire in *Camelus dromedarius* as in *Camelus ferus* and *Camelus bactrianus* with a great sequence identity between orthologous genes (Antonacci et al., 2017a; Antonacci et al., 2017b; Antonacci et al., 2019) is not shaped by SHM and it might be related to the constraint imposed on αß CDR1 and CDR2 domains by the requirements for binding to MH molecules, which, in turn, show a low level of genetic diversity in all three camel species (Plasil et al., 2016).

In Old World camels, the MHC region is structured similarly to a number of other mammalian species. Phylogenetic relationships of camel *MHC* genes do not always follow relationships based on other (neutral) nuclear genes. The diversity of the MH class I, II, and class I – related genes is generally lower than expected. This observation is consistent with a low genome-wide nuclear diversity in dromedaries, wild and domestic Bactrian camels (Fitak et al., 2016a; Fitak et al., 2016b). Several bottlenecks, in the evolutionary history of the three species, but also in recent times due to domestication of dromedaries and Bactrian camels or hunting and habitat decline of the wild camels, respectively, might be responsible for the reduced immunogenetic and genome-wide variability in Old World camels. However, experimental evidence for answering the question whether the low MHC diversity is really due to low diversity of the camel genomes is still lacking.

As the camel is a useful and promising model for therapeutic applications and for phylogenetic and evolution studies about the humoral and cell mediated immunity in jawed vertebrates, implementation of the camelid genomic sequences of IG, TR, and MHC loci is necessary to encourage progress for improvement of the global knowledge of the adaptive immune responses of these animal models.

## Author Contributions

SC, PB, SM, and RA designed and wrote the review; MP and PH contributed to manuscript writing; EC, VC, and GL contributed to searching of genomic literature. All authors have read and approved the final manuscript.

## Funding

Austrian Science Fund (FWF): P29623-B25.

## Conflict of Interest

The authors declare that the research was conducted in the absence of any commercial or financial relationships that could be construed as a potential conflict of interest.

## Footnotes

1
http://www.imgt.org
