# Supplementary figures and images for: The Camel Adaptive Immune Receptors Repertoire as a Singular Example of Structural and Functional Genomics
Source: Front Genet. 2019 Oct 17;10:997. doi: 10.3389/fgene.2019.00997 (PMC6812646; doi:10.3389/fgene.2019.00997)

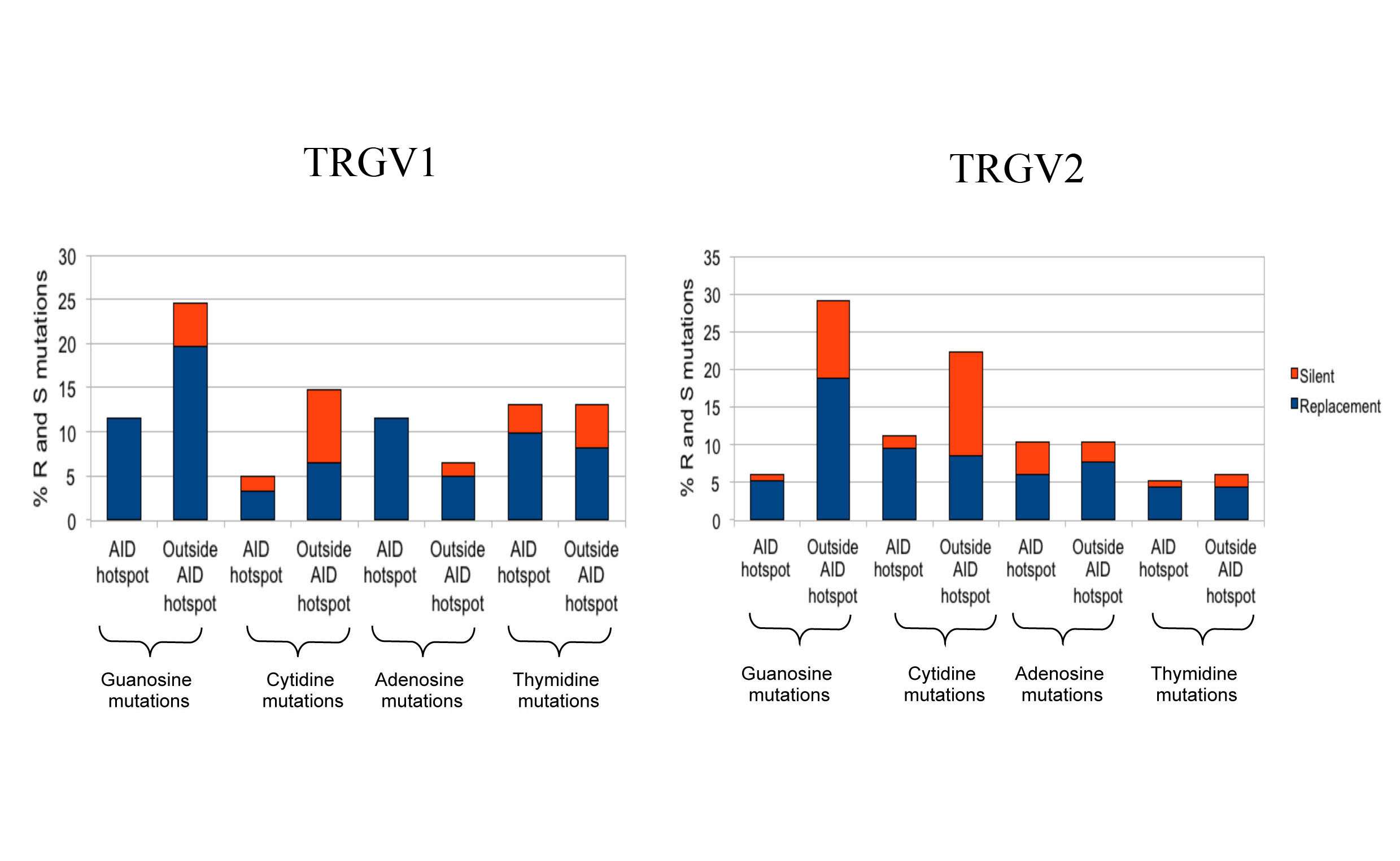

Supplement: Supplementary file 1 [file Image_1.jpeg]
